# Urothelial carcinoma of the bladder with prostatic stromal invasion and early postoperative bone metastases: a case report

**DOI:** 10.3389/fruro.2026.1910163

**Published:** 2026-08-21

**Authors:** Cheng Wang, Mengyun Wang, Yue He

**Affiliations:** 1 School of Clinical Medicine, North Sichuan Medical College, Nanchong, Sichuan, China; 2 Department of Urology, Suining Central Hospital, Suining, Sichuan, China

**Keywords:** bone metastases, case report, immunohistochemistry, prostatic stromal invasion, urothelial carcinoma of the bladder

## Abstract

**Background:**

Prostatic involvement by bladder urothelial carcinoma is uncommon, and prostatic stromal invasion usually indicates aggressive tumor biology and poor prognosis. Such patients may present with prostatic enlargement or a mass at the bladder neck-prostate junction, while serum prostate-specific antigen (PSA) may remain within the normal range. This diagnostic overlap may lead to clinical confusion with benign prostatic hyperplasia or primary prostatic tumors.

**Case presentation:**

A 54-year-old man was admitted with urinary frequency, urgency, dysuria, and intermittent gross hematuria. Serum PSA on admission was 2.1 ng/mL. Imaging suggested a mass at the bladder neck-prostate junction with an indistinct boundary between the bladder and prostate. After transurethral resection of a bladder lesion, pathology and immunohistochemistry indicated high-grade invasive urothelial carcinoma. Tumor cells expressed CK20, GATA3, and p63, partially expressed CK7 and Uroplakin III, and were negative for PSA and NKX3.1. The patient subsequently underwent radical cystectomy, ileal conduit urinary diversion, and pelvic lymph-node dissection. Postoperative pathology revealed high-grade bladder urothelial carcinoma with invasion of the prostatic stroma and capsule, staged as pT4aN0M0, with lymphovascular invasion, perineural invasion, and a positive prostatic urethral margin. PD-L1 expression was low (TPS <1%, IC <1%), and HER2 was negative. Because no neoadjuvant systemic therapy had been given and the final pathology showed pT4a disease with a positive margin, adjuvant systemic therapy was recommended and initiated. The patient received only approximately one week of platinum-based chemotherapy plus immunotherapy; treatment was discontinued because of poor tolerance and substantial financial burden. Three months after surgery, the patient developed low back pain. Whole-body bone scintigraphy and SPECT/CT demonstrated multiple bone metastases involving the L2 and L4 vertebrae and posterior elements, bilateral iliac bones, the left acetabular rim, and the right inferior pubic ramus. He subsequently received denosumab combined with conformal intensity-modulated radiotherapy to vertebral metastatic lesions.

**Conclusion:**

This case highlights that a normal PSA level does not exclude prostatic involvement by bladder-derived urothelial carcinoma. For patients with bladder neck or prostatic masses, especially those with normal PSA but imaging evidence of bladder-prostate junction involvement, an immunohistochemical panel including GATA3, CK7, CK20, p63, Uroplakin III, PSA, and NKX3.1 should be used early for differential diagnosis. Prostatic stromal invasion combined with lymphovascular invasion, perineural invasion, and a positive surgical margin may indicate highly aggressive disease and an increased risk of early distant metastasis, requiring intensified postoperative risk assessment, systemic treatment decision-making, and close follow-up.

## Introduction

Urothelial carcinoma of the bladder is one of the most common malignancies of the urinary system, and patients with locally advanced disease may develop prostatic involvement. According to the pattern of involvement, prostatic involvement may present as urethral mucosal involvement, prostatic ductal involvement, or prostatic stromal invasion. Among these patterns, prostatic stromal invasion is closely associated with greater tumor aggressiveness and poorer prognosis and is classified as pT4a disease in the AJCC TNM staging system.

In clinical practice, the diagnosis of bladder urothelial carcinoma involving the prostate often lacks specific clinical signs. When a lesion is located at the bladder neck-prostate junction and the serum PSA level is normal, the clinical and imaging findings may overlap with those of benign prostatic hyperplasia or primary prostate cancer, potentially delaying diagnosis and complicating treatment decisions.

The uniqueness of this case is reflected in three aspects. First, the patient had a normal serum PSA level, but the lesion was ultimately identified as high-grade urothelial carcinoma of bladder origin with prostatic stromal invasion. Second, an immunohistochemical panel was critical for excluding a prostatic origin and confirming urothelial differentiation. Third, although no pelvic lymph-node metastasis was identified, multiple bone metastases developed shortly after surgery, suggesting that prostatic stromal invasion combined with lymphovascular invasion, perineural invasion, and a positive margin may represent highly aggressive tumor biology. We report this case to improve recognition of prostatic stromal invasion by bladder-derived urothelial carcinoma in the setting of normal PSA and to discuss its diagnosis, prognosis, and postoperative management.

## Case presentation

### Initial presentation

A 54-year-old man was admitted to the Department of Urology of our hospital approximately two weeks before radical cystectomy with a history of urinary frequency, urgency, and dysuria for more than 3 months, accompanied by gross hematuria for 20 days. More than 3 months before admission, he developed urinary frequency, urgency, dysuria, and difficulty voiding but did not seek medical attention. Twenty days before admission, he began to experience intermittent gross hematuria throughout urination without blood clots or flank pain. Ultrasonography performed at an outpatient clinic of another hospital suggested a space-occupying bladder lesion. Since disease onset, the patient had maintained acceptable sleep and appetite, with normal bowel movements and no obvious weight loss. He had no significant past medical history.

On admission, physical examination revealed a flat, soft abdomen without tenderness. There was no percussion tenderness over either renal region, no tenderness along the ureteral course, and no suprapubic dullness to percussion. Digital rectal examination revealed an enlarged prostate with a soft consistency, a smooth surface, no obvious nodules or tenderness, and a shallow median sulcus; no blood staining was observed on the examining glove.

### Diagnostic assessment

**Figure 1:**
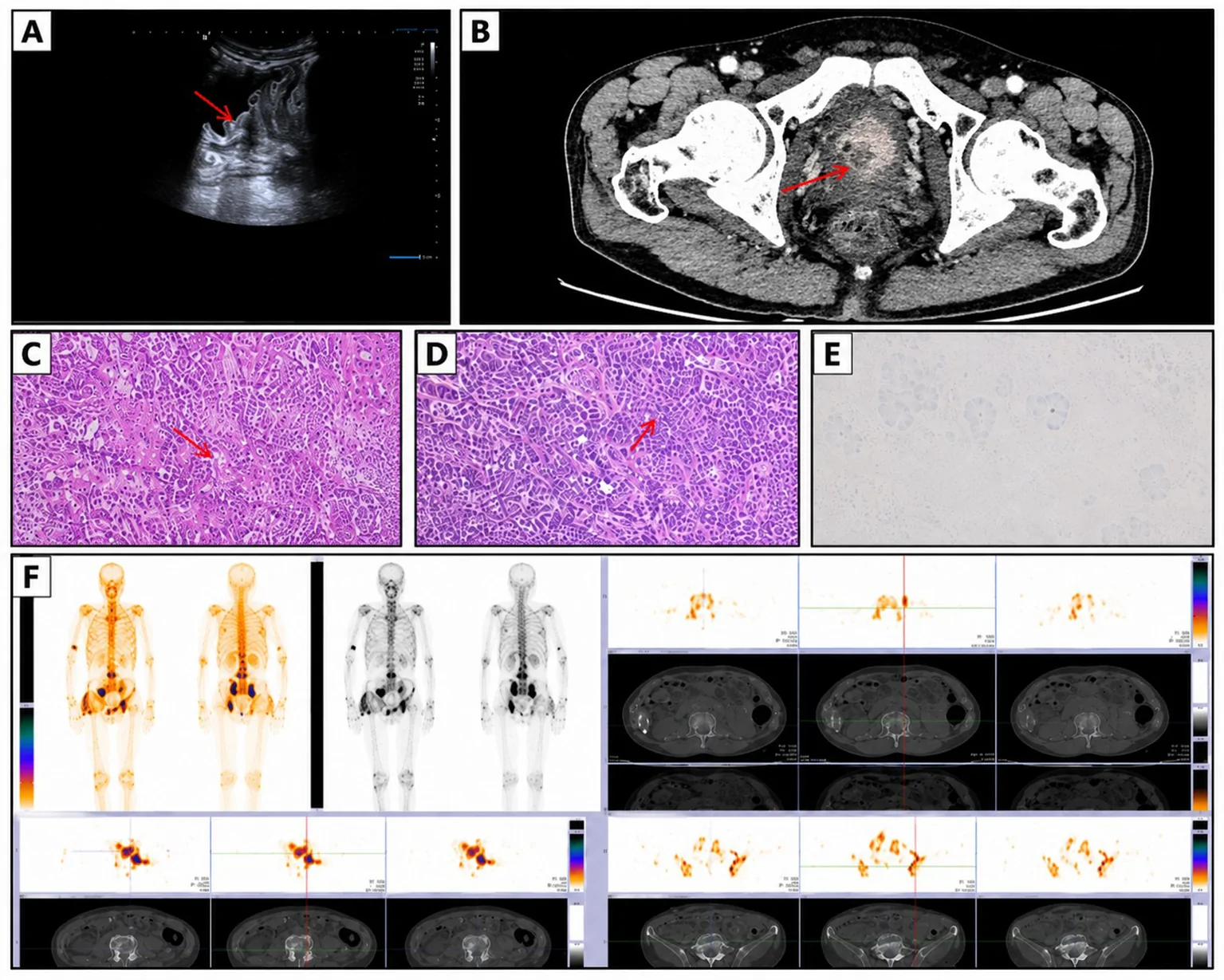
Diagnostic, pathological, and metastatic assessment. **(A)** Preoperative urinary ultrasonography showing a small protruding bladder lesion requiring differentiation between a bladder-derived and a prostate-derived lesion. **(B)** Preoperative contrast-enhanced CT showing patchy and nodular irregular enhancement, with suspected marked mucosal enhancement at the bladder base. **(C)** Pathology of the transurethral resection specimen showing atypical cells, supporting invasive high-grade urothelial carcinoma. **(D)** Pathology of the radical cystectomy specimen supporting invasive high-grade bladder urothelial carcinoma with prostatic involvement. **(E)** PD-L1 immunohistochemistry showing low expression (TPS <1%, IC <1%). **(F)** Whole-body bone scintigraphy and SPECT/CT fusion imaging showing multiple bone metastases.

Ultrasonography of the urinary system showed a small protruding lesion in the bladder, requiring differentiation between a bladder-derived and a prostate-derived lesion, and also demonstrated prostatic enlargement with calcifications (**Figure 1A**). Non-contrast and contrast-enhanced CT of the urinary tract showed poor bladder filling, an indistinct bladder-prostate boundary, and irregular morphology. Contrast-enhanced imaging revealed patchy and nodular irregular enhancement, with suspected marked mucosal enhancement at the bladder base. The prostate was enlarged and showed heterogeneous enhancement (**Figure 1B**). CT urography showed no abnormalities in renal excretion or bilateral ureteral visualization, while bladder contrast filling remained poor. The serum PSA level was 2.1 ng/mL (reference range, 0–4 ng/mL).

**Figure 2:**
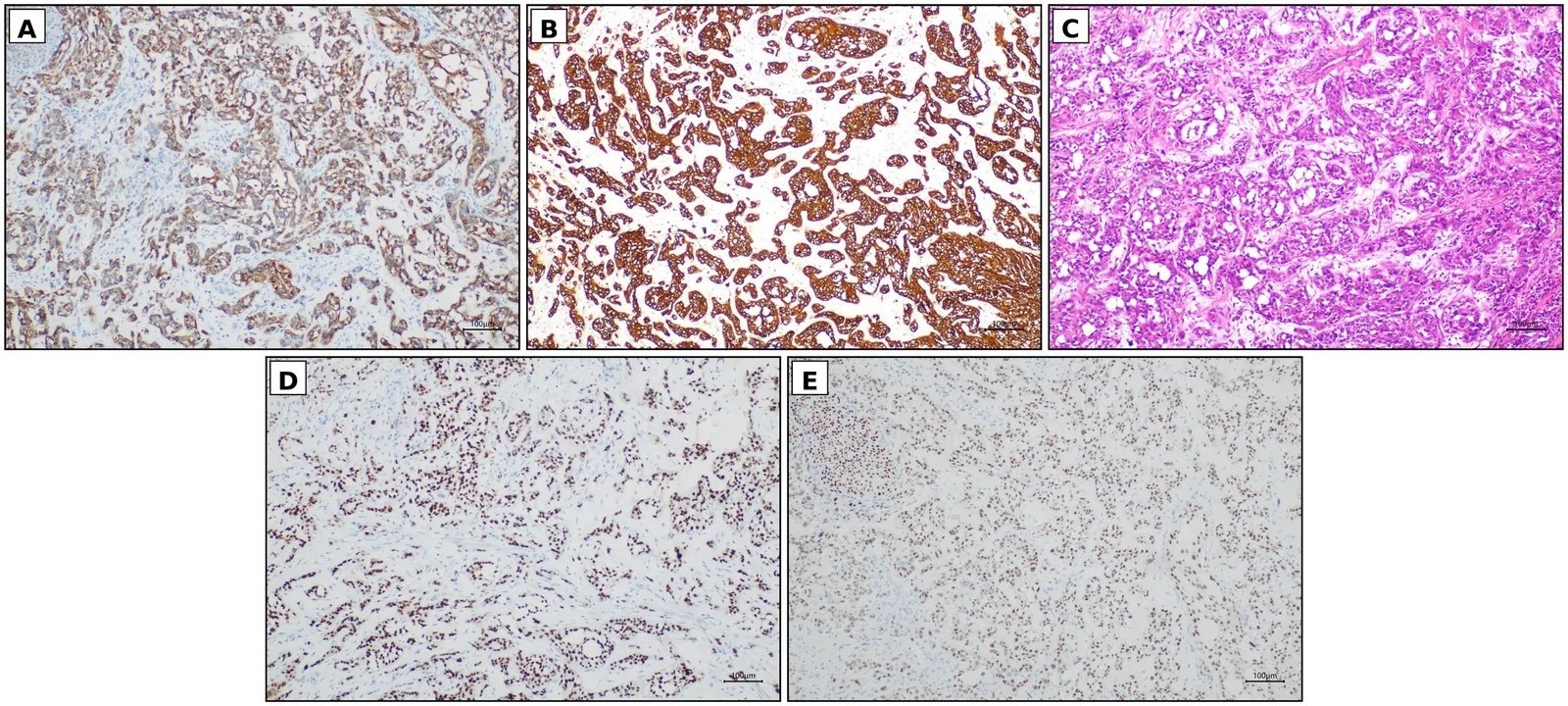
Histomorphologic and immunohistochemical findings supporting urothelial differentiation. **(A)** CK7 shows partial membranous and cytoplasmic positivity in tumor cells. **(B)** Pan-cytokeratin (CKpan/AE1/AE3) shows diffuse strong membranous and cytoplasmic staining. **(C)** Hematoxylin and eosin staining shows infiltrative high-grade carcinoma arranged in irregular nests and cords within desmoplastic stroma. **(D)** GATA3 shows strong nuclear positivity in tumor cells. **(E)** p63 shows nuclear positivity in tumor cells. Scale bars, 100 μm.

Following admission, transurethral resection of the bladder lesion was performed. Intraoperatively, mucosal hyperplasia and focal follicle-like changes were observed in the bladder trigone, along with a neoplasm involving the bladder neck and prostate. Histopathological examination showed a proliferative urothelial lesion in the bladder trigone and atypical cells in the neoplasm involving the bladder neck and prostatic region (**Figure 1C**). Immunohistochemistry showed CK(AE1/AE3) (+), CK20 (+), CK7 (partially +), GATA3 (+), HER2 (neo) (0), Ki-67 (+, approximately 70%), NKX3.1 (-), p63 (+), PSA (-), S100P (+), and Uroplakin III (partially +). Based on morphology and immunohistochemistry, the diagnosis was invasive high-grade urothelial carcinoma, clinically considered to originate from the bladder neck and invade the prostate. Representative histomorphologic and immunohistochemical photomicrographs are shown (**Figure 2**). H&E staining demonstrated infiltrative high-grade carcinoma arranged in irregular nests and cords within desmoplastic stroma. CK7 showed partial membranous and cytoplasmic positivity, while pan-cytokeratin (CKpan/AE1/AE3) showed diffuse strong staining, confirming epithelial differentiation. Nuclear GATA3 and p63 positivity, interpreted together with the documented PSA and NKX3.1 negativity, supported urothelial differentiation rather than a prostatic origin.

### Treatment and postoperative pathology

Two weeks after admission, the patient underwent radical cystectomy, ileal conduit urinary diversion, and pelvic lymph-node dissection under general anesthesia and was discharged uneventfully 10 days after surgery. Postoperative pathology showed invasive high-grade urothelial carcinoma of the bladder, with a tumor size of approximately 4.5 cm × 4.0 cm × 1.0 cm. The tumor invaded the full thickness of the bladder wall and involved the prostatic stroma and capsule; lymphovascular and perineural invasion were present (**Figure 1D**). Histologic examination demonstrated direct, contiguous extension of the bladder primary into the prostatic stroma and capsule; this route of invasion formed the basis for the pT4a assignment. No carcinoma was found in the bilateral seminal vesicles, vas deferens, or ureteral margins. The distal prostatic urethral margin was positive, but this was recorded separately as a margin-status finding and did not affect the pT4a stage. A total of 14 pelvic lymph nodes (8 left and 6 right) were negative for carcinoma. Postoperative immunohistochemistry showed CD45 (LCA) (-), CK(AE1/AE3) (+), and GATA3 (partially +). Combined with the histological morphology and immunohistochemical findings, these results supported urothelial carcinoma and excluded a prostatic tumor origin. The pathological stage was pT4aN0M0.

To guide subsequent adjuvant therapy, PD-L1 immunohistochemistry was performed on the primary lesion. The tumor proportion score (TPS) was <1%, and the immune-cell score (IC) was <1%; therefore, PD-L1 expression was interpreted as low (**Figure 1E**).

Postoperative systemic-therapy decision-making was revisited after multidisciplinary discussion. Because the patient had high-risk features including pT4a disease, lymphovascular invasion, perineural invasion, and a positive distal prostatic urethral margin, we recognized that perioperative/adjuvant systemic treatment was indicated. Current guideline-based options support adjuvant cisplatin-based combination chemotherapy for patients with pT3/4 and/or pN+ disease when no neoadjuvant systemic therapy has been given, while adjuvant nivolumab is a recommended option for high-risk muscle-invasive urothelial carcinoma when cisplatin-based adjuvant chemotherapy is not feasible or is declined (1, 2).

In this context, adjuvant systemic therapy was recommended. The planned cisplatin-based chemotherapy schedule was gemcitabine 1,000 mg/m² intravenously on days 1 and 8 plus cisplatin 70 mg/m² intravenously on day 1, repeated every 21 days for up to four cycles if tolerated (3). Adjuvant nivolumab was discussed as the immunotherapy option, with a commonly used schedule of 240 mg intravenously every 2 weeks or 480 mg intravenously every 4 weeks until recurrence, unacceptable toxicity, or up to 1 year. In actual clinical practice, the patient received only approximately one week of platinum-based chemotherapy plus immunotherapy. Treatment was then discontinued after renewed shared decision-making because of poor tolerance and substantial financial burden. We therefore acknowledge that the inability to deliver complete perioperative/adjuvant systemic therapy is a major limitation of this case and an important context for interpreting the subsequent early bone metastases.

### Early postoperative bone metastases, treatment, and follow-up

Approximately three months after radical cystectomy, the patient visited the Department of Oncology of our hospital because of low back pain. Whole-body bone scintigraphy and SPECT/CT fusion imaging showed multiple osteolytic bone metastases involving the L2 and L4 vertebrae and posterior elements, bilateral iliac bones, the left acetabular rim, and the right inferior pubic ramus, consistent with multiple bone metastases from bladder cancer (**Figure 1F**).

Soon after the diagnosis of bone metastases, the patient received denosumab 120 mg by subcutaneous injection every 4 weeks to reduce the risk of skeletal-related events. At the same time, intensity-modulated radiotherapy was delivered to the vertebral metastatic lesions at a prescribed dose of 30 Gy in 10 fractions (DT 3000 cGy). The patient is currently continuing treatment and follow-up.

## Discussion

Urothelial carcinoma involving the prostate can be classified as primary or secondary. Primary prostatic urothelial carcinoma is rare, accounting for only 1%-4% of prostate malignancies (4), and mainly originates from the transitional epithelium of the prostatic urethra or prostatic ducts (5). In contrast, secondary prostatic involvement is most commonly caused by bladder urothelial carcinoma spreading along the urethral mucosa, through prostatic ducts, or by direct invasion; it has been reported in approximately 12%-40% of radical cystectomy specimens (6). Studies of radical cystoprostatectomy specimens have shown that prostatic involvement may occur at different levels, including the mucosa, ducts, and stroma; among these, stromal invasion reflects aggressive tumor biology more accurately than the extent of superficial involvement alone (7). Prostatic stromal invasion is regarded as an important pathological feature of aggressive disease and has been classified as pT4a disease in the AJCC TNM staging system (8). From a molecular perspective, urothelial carcinoma is characterized by a highly heterogeneous genomic landscape involving alterations in pathways related to chromatin remodeling, DNA damage repair, cell-cycle regulation, and receptor tyrosine kinase signaling. Comprehensive genomic analyses have demonstrated that muscle-invasive bladder cancer frequently harbors alterations in TP53, RB1, ERCC2, FGFR3, PIK3CA, ARID1A, and other chromatin remodeling genes, which contribute to genomic instability, tumor progression, and biological heterogeneity (9, 10). Recent molecular classification studies have further divided muscle-invasive bladder cancer into distinct molecular subtypes, including basal/squamous and luminal subtypes, which demonstrate different biological characteristics and prognostic implications. Basal/squamous tumors are frequently associated with TP53/RB1 pathway alterations and aggressive phenotypes, whereas luminal tumors often exhibit urothelial differentiation signatures and FGFR pathway alterations (11). Although comprehensive genomic sequencing was not performed in this patient, the rapid postoperative metastatic progression and extensive pathological risk factors may indicate underlying molecular abnormalities associated with aggressive tumor behavior. Future genomic profiling may help clarify the mechanisms responsible for prostatic stromal invasion and early distant dissemination in this rare subgroup. The most clinically valuable feature of this case is the diagnostic pitfall. The patient presented mainly with urinary frequency, urgency, dysuria, and gross hematuria, accompanied by prostatic enlargement, but the PSA level was normal, making the condition easy to misdiagnose as benign prostatic hyperplasia or primary prostate cancer. Previous studies have shown that PSA is often normal or only mildly elevated when bladder-derived urothelial carcinoma involves the prostate; therefore, a normal serum PSA level cannot be used to exclude urothelial carcinoma involving the prostate (12). In addition, imaging in this case showed an indistinct boundary between the bladder neck and prostate and irregular enhancing foci, consistent with previously reported imaging features of locally advanced bladder cancer (13).

Immunohistochemical analysis was central to the correct diagnosis in this case. The tumor cells expressed CK20, GATA3, and p63, partially expressed CK7 and Uroplakin III, and were negative for PSA and NKX3.1, consistent with a typical immunophenotype of urothelial carcinoma. GATA3 is an important marker of urothelial differentiation and has high sensitivity in high-grade urothelial carcinoma (14), whereas NKX3.1 is considered a sensitive and specific marker of prostatic origin (15). However, some high-grade prostatic adenocarcinomas may exhibit solid, nested, or pseudopapillary growth patterns and can closely mimic urothelial carcinoma morphologically, particularly in tumors involving the bladder neck or prostatic urethra. Therefore, careful correlation with immunohistochemical findings is required to establish the correct tumor origin (16). Therefore, a single immunomarker is insufficient for reliably distinguishing between these two tumor types. Previous studies support the combined use of urothelial markers, such as GATA3, p63, and Uroplakin III, and prostatic markers, such as PSA and NKX3.1, in the differential diagnosis of high-grade tumors involving the bladder neck and prostatic region (17, 18). Beyond immunophenotypic evaluation, accurate pathological diagnosis requires careful assessment of tumor morphology and the anatomical pattern of prostatic involvement. In bladder urothelial carcinoma involving the prostate, tumor extension may occur through three major pathways: prostatic urethral mucosal involvement, prostatic ductal involvement, and direct stromal invasion. Among these patterns, stromal invasion represents the most clinically significant form because it reflects tumor infiltration into the prostatic parenchyma and is associated with advanced pathological stage and poorer oncological outcomes (7, 13). Histologically, urothelial carcinoma involving the prostate may demonstrate nested, solid, or infiltrative growth patterns, which can overlap with poorly differentiated prostatic adenocarcinoma. Therefore, morphological evaluation alone may be insufficient, particularly in limited biopsy specimens. High-grade prostatic adenocarcinoma may occasionally demonstrate pseudopapillary, nested, or solid growth patterns, resulting in morphological similarity to urothelial carcinoma and representing an important diagnostic pitfall (16). Therefore, the diagnosis of bladder urothelial carcinoma with prostatic involvement should integrate histological morphology, clinical presentation, imaging findings, serum PSA levels, and immunohistochemical profiles. A combined panel of urothelial markers (GATA3, p40/p63, CK20, Uroplakin II/III) and prostate lineage markers (PSA and NKX3.1) provides higher diagnostic accuracy than any single marker alone (17, 18).

For masses located at the bladder neck or prostate, especially in patients with normal serum PSA, a routine immunohistochemical panel including GATA3, CK7, CK20, p63, PSA, and NKX3.1 is recommended to improve diagnostic accuracy.

Another important implication of this case is the possible association between prostatic stromal invasion and early postoperative distant metastasis. Existing studies have confirmed that prostatic stromal invasion is an important adverse prognostic factor in patients with bladder cancer. Shen et al. retrospectively analyzed 89 patients with bladder cancer and prostatic involvement and found that prostatic stromal invasion was closely associated with decreased cancer-specific and overall survival and represented an independent adverse prognostic indicator (4). Moschini et al. further demonstrated that, compared with mucosal or ductal involvement alone, prostatic stromal invasion was associated with a higher risk of local recurrence and distant metastasis (13). Similarly, Barocas et al. found that the depth of prostatic involvement was closely related to prognosis and that prostatic stromal invasion independently predicted poor overall survival (19). Kiyoshima et al. further indicated that prognosis is determined primarily by the presence of stromal invasion rather than by whether prostatic involvement is contiguous or non-contiguous (20). Together, these findings suggest that prostatic stromal invasion should not be viewed merely as an extension of local anatomical involvement, but rather as an important pathological indicator for assessing the risk of postoperative recurrence and distant metastasis. In the present case, although pelvic lymph-node metastasis was not detected, multiple bone metastases developed approximately 3 months after surgery, suggesting that occult micrometastases not detectable on routine preoperative imaging may already have been present. Lymphovascular invasion, perineural invasion, and a positive prostatic urethral margin further indicated highly aggressive tumor biology. Together, these factors may explain the rapid postoperative progression observed in this patient. However, because complete neoadjuvant or adjuvant systemic therapy was not delivered, the early bone recurrence should not be presented as a purely natural consequence of prostatic stromal invasion alone; rather, it should be interpreted as the combined effect of aggressive pathological risk factors and incomplete systemic disease control.

From a management perspective, radical cystoprostatectomy remains an important local treatment for muscle-invasive bladder cancer with prostatic stromal invasion; however, local treatment alone is frequently insufficient for high-risk disease. Current EAU guidance recommends perioperative chemoimmunotherapy with cisplatin/gemcitabine plus durvalumab or neoadjuvant cisplatin-based combination chemotherapy for eligible patients with T2-T4a, cN0-1, M0 muscle-invasive bladder cancer, and recommends adjuvant cisplatin-based combination chemotherapy for pT3/4 and/or pN+ disease if no neoadjuvant systemic therapy was given (1, 2). Adjuvant nivolumab is also recommended for high-risk muscle-invasive urothelial carcinoma (≥ypT2N0 after neoadjuvant chemotherapy or pT3/4 and/or pN+) when adjuvant cisplatin-based chemotherapy is not feasible or is declined; regulatory indications differ by jurisdiction, with FDA approval irrespective of PD-L1 status and EMA approval limited to tumors with PD-L1 tumor-cell expression ≥1% (21).

In the present case, neoadjuvant chemotherapy was not administered before cystectomy. At the time of initial treatment, the lesion was considered operable after transurethral resection and diagnostic confirmation, the patient had bothersome urinary symptoms and wished to proceed rapidly to definitive surgery, and the final pathological risk profile (pT4a disease with a positive prostatic urethral margin, lymphovascular invasion, and perineural invasion) was not fully established until radical cystectomy. In retrospect, we agree that this management pathway deviated from the preferred systemic-treatment strategy for high-risk muscle-invasive urothelial carcinoma. After the final pathology became available, adjuvant systemic therapy was recommended. The patient initiated platinum-based chemotherapy plus immunotherapy but discontinued treatment after approximately one week because of poor tolerance and financial burden. Therefore, the subsequent early bone metastases may reflect both aggressive tumor biology and inadequate systemic treatment exposure. A phase III randomized controlled trial showed that denosumab was non-inferior to zoledronic acid in delaying skeletal-related events in patients with bone metastases from advanced solid tumors and was associated with a lower incidence of renal toxicity (22). However, in patients with symptomatic bone metastases, antiresorptive therapy alone is often insufficient to control local pain and maintain skeletal stability, and local radiotherapy is usually required as part of comprehensive treatment. ASTRO guidelines indicate that palliative radiotherapy can effectively relieve pain associated with bone metastases and help achieve local lesion control (23). Accordingly, the present patient received denosumab combined with local radiotherapy to vertebral lesions, with the aim of reducing the risk of skeletal-related events and improving quality of life.

## Conclusion

For patients with a normal serum PSA level but a mass at the bladder neck or prostatic region, clinicians should remain alert to the possibility of prostatic involvement by bladder-derived urothelial carcinoma. This case suggests that an immunohistochemical panel is central to distinguishing bladder-derived urothelial carcinoma from primary prostatic tumors. Prostatic stromal invasion combined with lymphovascular invasion, perineural invasion, and a positive surgical margin may indicate highly aggressive disease and an increased risk of early distant metastasis. Such patients require more active postoperative risk assessment, systemic treatment decision-making, and close follow-up. When perioperative or adjuvant systemic therapy cannot be administered or completed, any deviation from the recommended treatment plan should be thoroughly documented, discussed with the patient, and taken into consideration when interpreting early recurrence. In this case, the early bone metastases are therefore best interpreted as a consequence of both aggressive pathological risk factors and the incomplete delivery of systemic therapy, rather than as a novel diagnostic entity.

## Data Availability

The original contributions presented in this case report are included in the article. Further inquiries can be directed to the corresponding author.
